# Glycemic Control During Enteral Tube Feeding in Patients with Diabetes Mellitus

**DOI:** 10.7759/cureus.3929

**Published:** 2019-01-21

**Authors:** Benedicta N Sarfo-Adu, Jemma L Hendley, Barbara Pick, Samson O Oyibo

**Affiliations:** 1 Internal Medicine, Peterborough City Hospital, Peterborough, GBR

**Keywords:** enteral tube feeding, glycaemic control, diabetes, nutrition, dietitian, diabetes specialist nurse

## Abstract

Introduction

Achieving good glycemic control during enteral tube feeding in patients with diabetes mellitus can be difficult. National guidelines emphasize the need for the early involvement of the dietitian and diabetes specialist nurse, regular capillary blood glucose (CBG) monitoring, and the avoidance of hypoglycemic events. We aimed to assess glycemic control in patients with diabetes during enteral tube feeding.

Materials and methods

A retrospective study, involving patients with diabetes who had enteral tube feeding during their hospital stay from January to December 2016, was performed. Data included feed carbohydrate content, infusion rate and duration, involvement of dietitian and inpatient diabetes specialist nurse, capillary blood glucose monitoring, and documentation of hypoglycemic events.

Results

There were 40 patient episodes. Mean (range) age: 67 (29-94) years, 60% were male, 97.5% had type 2 diabetes, and 60% were on oral hypoglycemic agents prior to admission. The average feed carbohydrate content was 14.6 g/dL and the average feed rate was 73 ml/hour. Dietitians and diabetes inpatient specialist nurses (DISNs) were involved in 100% and 75% of cases, respectively. During enteral tube feeding, capillary blood glucose was controlled using metformin, subcutaneous insulin, or intravenous insulin in 30%, 42.5%, and 15% of cases, respectively. Capillary blood glucose was checked four to six hourly in 100% of cases. The target capillary blood glucose range (6-12 mmol/L) was achieved in 40% of cases. The minimum capillary blood glucose value (median (interquartile range)) was 4.9 (3.9-6.2) mmol/L and the maximum value was 15.1 (11.9-18.8) mmol/L. Using these lower and upper quartile values for minimum and maximum values, respectively, revealed that 57.5% of patients had a capillary blood glucose range of 3.9-18.8 mmol/L. Two patients had hypoglycemic events requiring treatment.

Conclusions

This study demonstrated that despite adequate adherence to our trust's guidelines for glycemic control during enteral tube feeding in patients with diabetes, the target glycemic range was achieved in 40% of cases. The importance of the early involvement of the diabetes inpatient specialist nurse cannot be overemphasized. Early initiation and proactive (daily) dose up-titration of insulin are required to improve glycemic control during enteral tube feeding. A national audit tool for glycemic control and mortality data during enteral tube feeding is required.

## Introduction

Patients who cannot swallow for clinical reasons (e.g., stroke, reduced consciousness, undernutrition, recovery from surgery or trauma, etc.) are put on enteral tube feeding (ETF) to cater to their metabolic needs. The feed consists of a mixture of carbohydrates, protein, fat, minerals, vitamins, and water in compositions depending on individual need and as determined by the dietitian [1].

Achieving optimal glycemic control during ETF in patients with diabetes can be difficult because the enteral feed provides a continuous infusion of glucose (10-20 grams of carbohydrates per hour), which is different from normal eating patterns [2]. Additionally, the patient’s usual oral anti-diabetic medications cannot be administered down the tube because of the risk of tube blocking and drug-formula incompatibilities [3].

Before national guidelines were produced, we prospectively followed 24 patients who had a stroke and required ETF. We demonstrated that a simple, twice daily insulin regimen safely provided adequate glycemic control without hypoglycemic episodes in a majority of patients as long as frequent blood glucose monitoring was carried out [4]. The Joint British Diabetes Societies (JBDS) guidelines were produced in 2012 for glycemic management during ETF of people with stroke and diabetes [5]. These guidelines emphasized the importance of achieving a safe target glucose range (6-12 mmol/L) without causing significant hypoglycemia, early involvement of the dietitian and diabetes inpatient specialist nurse (DISN), monitoring capillary blood glucose (CBG) four to six hourly, and continuing long-acting basal insulin. It also suggested insulin regimens for glycemic control and mentioned the use of metformin for mild hyperglycemia in patients with type 2 diabetes [5-6].

We updated our Trust guidelines following the introduction of the JBDS guidelines in 2012 and used them for all patients who had diabetes and required ETF because of a stroke or any other medical reason. Therefore, the aim of this project was to assess glycemic control in patients with diabetes during ETF.

## Materials and methods

Patient group

This was a retrospective study looking at patients who had a diagnosis of diabetes mellitus and required enteral tube feeding (nasogastric, nasojejunal, or percutaneous endoscopic gastrostomy) during their hospital stay from January to December of 2016. Clinical coding was used to provide a list of patients.

Data collection

Patients who were known to be on ETF that attended hospital for a day case procedure were not included in this audit. Data included the feed carbohydrate content, the infusion rate, and the feeding duration over a 24-hour period, whether there was involvement of the dietitian and DISN. Evidence of CBG monitoring and the CBG levels were assessed along with the documentation of any hypoglycemic events (CBG value less than 4 mmol/L) requiring treatment. Data concerning demographics and clinical information were obtained from our hospital clinical notes database. Data concerning CBG results were obtained from our CBG data storage system. Data were collected over six months.

This study was deemed exempt from ethical approval on account of it being registered with our Quality, Governance, and Compliance department as a service improvement project.

## Results

We had 66 patient notes of which 26 were excluded because enteral feeding was never started for clinical reasons, such as the feeding tube did not stay in, the patient’s swallowing improved, the patient was too unwell to start feeding, or the patient refused feeding tube insertion. Therefore, 40 patient notes were examined. Table 1 shows the demographics, the diabetes treatment before ETF, and clinical reasons for requiring ETF.

**Table 1:** Demographics for the patient group *The reason for requiring enteral tube feeding was not recorded for five patients.

Sample size	40
Age (mean (range) years)	67 (29-94)
Gender (Male/Female)	24/16
Type of diabetes (Type 2/Type 1)	39/1
Anti-diabetic treatment regimen before admission and requiring enteral tube feeding	
Diet	7
Oral medication (Metformin, Gliclazide, Tolbutamide, Pioglitazone)	24
Insulin	8
Oral medication & insulin	1
Clinical reason for requiring enteral tube feeding*	
Stroke	11
Ventilatory support	9
Unsafe swallowing	9
Long-term percutaneous enterogastrostomy feeding	4
Eating disorder	2

Information concerning enteral tube feeding

Different types of feeds were used, containing between 12.3-20.1 grams of carbohydrates per 100 mL of feed. Patients were initiated at a low rate, and this was gradually increased to the maximum rate. The duration of feed ranged from four hours to 24 hours over a 24-hour period. This information is shown in Table 2.

**Table 2:** Information concerning the feed used for enteral tube feeding *Some patients had percutaneous endoscopic gastrostomy tube insertion and went home with this for long-term feeding.

	Average	Range
Carbohydrate content of the feed (gram/100mL)	14.59	12.3-20.1
Initial infusion rate of the enteral feed (mL/hour)	37.59	10-90
Final (maximum) infusion rate of the feed (mL/hour)	73.1	25-125
Final duration of enteral tube feeding over the 24 hours (hour)	16.13	4-24
Number of days on a stable feed infusion rate (days, excluding initial period of feed initiation)*	12.25	2-54

Treatments used for glycemic control

Various treatments were used to achieve glycemic control in this group of patients. Variable rate intravenous insulin infusion was used during the initial up-titration of the ETF for patients with type 1 diabetes and in patients with type 2 diabetes who had severe hyperglycemia. Once the patient was on a stable, ETF twice daily, premixed insulin regimen and was commenced according to the formula devised during our observational study in 2012 [4]. Over half of the patients had insulin treatment, of which twice daily premixed insulin was the main regimen in the majority of cases. Thirty percent of patients used metformin only; these were patients with type 2 diabetes or previously diet-controlled diabetes. These data along with the frequency of hypoglycemic events requiring treatment are shown in Table 3.

**Table 3:** Various treatments used to manage glycemic control during enteral tube feeding

Treatment	Number of patients	Frequency of hypoglycemic events requiring treatment
Metformin	12	0
Humulin-M3 insulin	14	2
Variable rate insulin infusion	6	0
Lantus, Levemir, Insulatard	3	0
No medication needed	5	0

Overall adherence to Trust guidelines

A dietitian was involved in all the cases and the DISN was involved in three-quarters of cases. When a twice daily mixed insulin regimen was used, the dose was calculated according to guidelines in all cases. CBG monitoring was performed adequately (four to six hourly) in all patients. Only two patients (5%) had hypoglycemic events requiring treatment.

Glycemic control during enteral tube feeding

**Figure 1:**
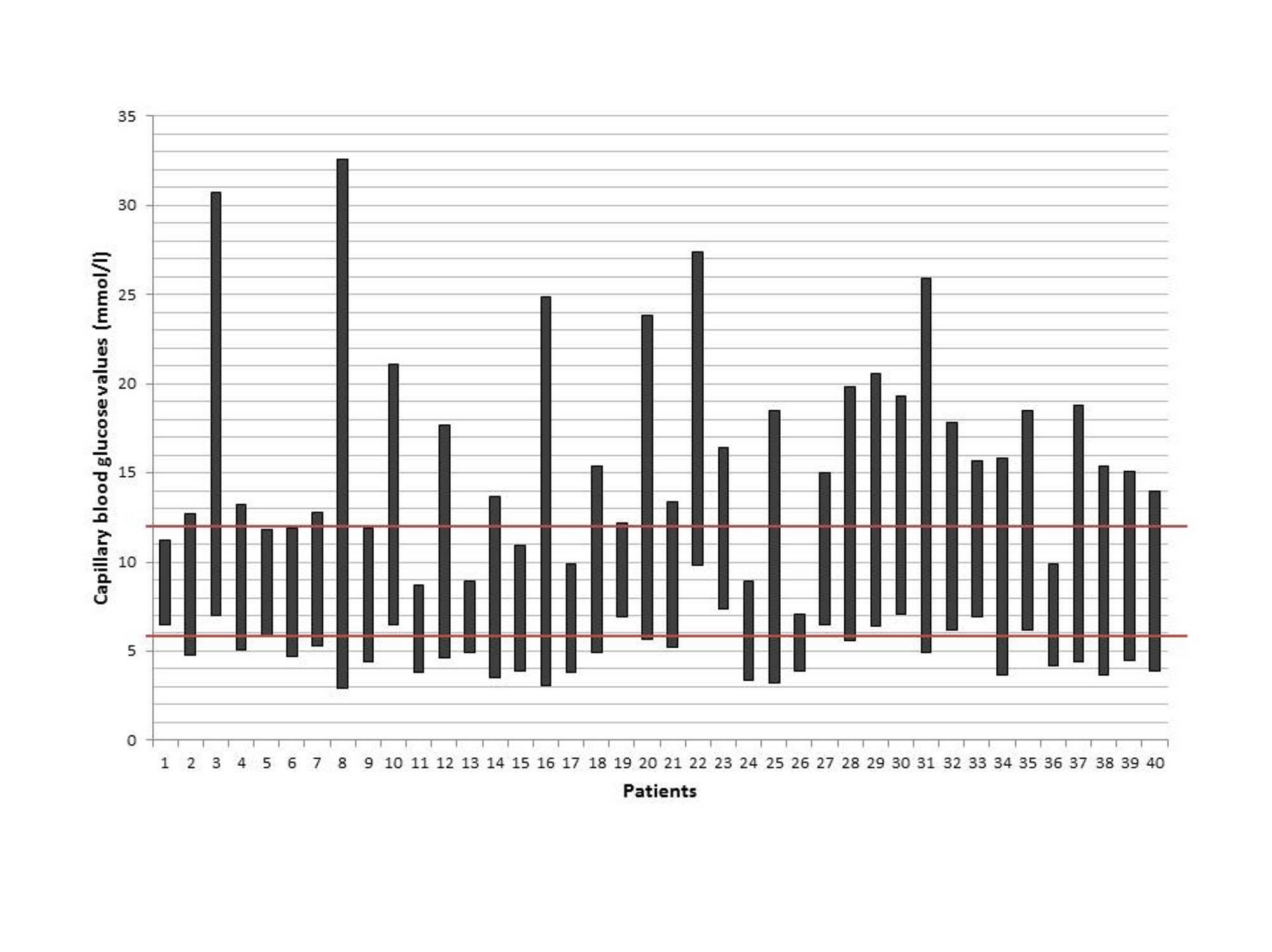
Graph showing glycemic control in relation to the target during enteral tube feeding for the 40 patients The vertical bars represent each patient’s capillary blood glucose readings during enteral tube feeding (ranging from minimum to maximum) in relation to the target capillary blood glucose range (represented by horizontal bars at the 6 mmol/l and 12 mmol/l points).

The target glycemic range of 6-12 mmol/L (±1 mmol/L) was achieved in 40% of patients during ETF. The minimum CBG value (median (interquartile range)) was 4.9 (3.9-6.2) mmol/L while the maximum CBG value (median (interquartile range)) was 15.1 (11.9-18.8) mmol/L. The individual CBG values (ranges) in relation to the recommended target range are shown in Figure 1.

Further analysis revealed that the recommended target glycemic range was achieved in eight of 12 patients (66%) who were on metformin during ETF in contrast to not achieving this in any of the patients that were on a twice-daily mixed insulin regimen. This was despite regular up-titration of the insulin dose. However, an examination of the first quartile for minimum CBG values demonstrated that 75% of all patients had a minimum CBG value of not less than 3.9 mmol/L. Conversely, an examination of the third quartile for maximum CBG values demonstrated that 75% of all patients in this study had a maximum CBG value of not more than 18.8 mmol/L. When we used these corresponding quartile values as our achievable range, we found that that 57.5% of individual patient CBG ranges fell within 3.9-18.8 mmol/L.

## Discussion

This study assessed glycemic control during ETF in patients with diabetes. Whilst demonstrating good adherence to our Trust guidelines, we revealed the inability to achieve the recommended target glycemic control in the majority of cases. However, the CBG range achieved for the majority of patients in this study was safe and accompanied by the avoidance of major hypoglycemic events requiring treatment. There is a scarcity of published data on glycemic control during ETF since the publication of the national (JBDS) guidelines. A previous similar study also highlighted how difficult it was to achieve the glycemic target range. Patients on ETF had suboptimal glucose control [2].

The national guidelines were published based on and specifically for patients with diabetes who had a stroke [5]. However, our study has demonstrated that the guidelines can be applicable to patients on ETF who have other medical or surgical causes for not being able to swallow or requiring increased nutrition. As in all patients, the risks and benefits of enteral tube insertion and feeding must be balanced with the clinical picture and the long-term prognosis of the patient.

Glycemic control during ETF is faced with a multitude of challenges, namely intercurrent illnesses and treatments, feed interruptions, and feed changes [7]. The patients in this study had multiple medical problems, including chest infections, acute kidney impairment, feed interruptions, and the use of steroid treatment. This may have contributed to the difficulties in achieving the CBG target range of 6-12 mmol/L on a twice-daily premixed insulin regimen.

The amount of carbohydrates, particularly glucose, being supplied to the patient during ETF is significant. A large systematic review demonstrated that the use of diabetes-specific formulas for ETF was associated with improved glycemic control compared with the standard formulas [8]. The diabetes-specific formulas had less carbohydrate content but more fat and fiber content compared to the standard formulas and, therefore, produced slower gastric emptying, slower nutrient assimilation, and less glucose rise [9]. Six years after the JBDS guidelines, several studies are still being performed to assess the optimal feed constituents and optimal insulin regimen to aid glycemic control during ETF [10-12]. These and ongoing studies substantiate the fact that glycemic control is difficult to achieve in patients with diabetes during ETF. The importance of early involvement of the DISN during ETF in patients with diabetes cannot be overemphasized [5-6].

Despite adequate adherence to guidelines, this study has its limitations. Firstly, this was a retrospective study with a small group of patients. Therefore, results may not be generalizable without larger studies. Secondly, we did not collect data concerning intercurrent illnesses, the use of steroids, or other causes of uncontrolled hyperglycemia. This data could have helped in elucidating reasons for the difficulty in achieving the target CBG control. Thirdly, due to a difficulty with clinical coding, we did not include patients who previously did not have diabetes. A prospective study is required to assess for ETF-induced hyperglycemia in patients who do not have pre-existing diabetes. Fourthly, retrospective data collection made it difficult to ascertain if glucose control was assessed while the feed rate was being increased or when patients were on a stable ETF and subcutaneous insulin regimen. Despite these limitations, this is one of the few published studies looking at glycemic control during ETF in patients with diabetes since the publication of the national guidelines.

## Conclusions

In conclusion, this study demonstrated adequate adherence to the JBDS guidelines for glycemic control during ETF in patients with diabetes. Despite this, it was still difficult to achieve the recommended target glycemic range. The results of this study emphasize the need to involve the DISN for all cases so that early and proactive initiation of subcutaneous insulin can occur. Additionally, if glycemic control is not fully achieved on metformin, subcutaneous insulin should be introduced. A JBDS national audit tool for assessing glycemic control and mortality data during ETF is required.

## References

[1] (2018). Nutritional support for adults: oral nutritional support, enteral tube feeding and parenteral nutrition. https://www.nice.org.uk/guidance/cg32.

[2] Murphy PM, Moore E, Flanagan DE (2014). Glycaemic control in insulin requiring diabetes patients receiving exclusive enteral tube feeding in an acute hospital setting. Diabetes Res Clin Pract.

[3] Williams NT (2008). Medication administration through enteral feeding tubes. Am J Health Syst Pharm.

[4] Oyibo SO, Sagi S, Home C (2012). Glycaemic control during enteral tube feeding in patients with diabetes who have had a stroke: a twice-daily insulin regimen. Practical Diabetes.

[5] Roberts A, Penfold S, Joint British Diabetes Societies (JBDS) for Inpatient Care Group (2018). Glycaemic management during the inpatient enteral feeding of stroke patients with diabetes. http://www.diabetologists-abcd.org.uk/JBDS/JBDS_IP_Enteral_Feeding_Stroke.pdf.

[6] Roberts AW, Penfold S, Joint British Diabetes Societies (JBDS) for Inpatient Care (2018). Glycaemic management during the inpatient enteral feeding of people with stroke and diabetes. Diabet Med.

[7] Richardson EA, Agbasi N (2017). Managing glycaemic trends in people with diabetes requiring enteral feeding support: the challenges in primary and secondary care. J Diabetes Nurs.

[8] Elia M. Ceriello A, Laube H, Sinclair AJ, Engfer M, Stratton RJ (2005). Enteral nutritional support and use of diabetes-specific formulas for patients with diabetes: a systematic review and meta-analysis. Diabetes Care.

[9] Coulson AM (1998). Clinical experience with modified enteral formulas for patients with diabetes. Clin Nutr.

[10] Robert S, Brody R, Rawal S, Byham-Gray L (2018). Volume-based vs rate-based enteral nutrition in the intensive care unit: impact on nutrition delivery and glycaemic control. J Parenter Enteral Nutr.

[11] Huhmann MB, Yamamoto S, Neutel JM, Cohen SS, Ichoa Gautier JB (2018). Very high-protein and low-carbohydrate enteral nutrition formula and plasma glucose control in adults with type 2 diabetes mellitus: a randomized crossover trial. Nutr Diabetes.

[12] Drincic AT, Knezevich JT, Akkireddy P (2017). Nutrition and hyperglycaemia management in the inpatient setting (meals on demand, parenteral, or enteral nutrition). Curr Diab Rep.

